# Molecular imaging and immune cell adhesion via vascular adhesion protein-1 in idiopathic inflammatory myopathy: a case report

**DOI:** 10.1016/j.ero.2025.04.005

**Published:** 2025-05-10

**Authors:** Simon M. Petzinna, Jim Küppers, Maike S. Adamson, Benedikt Schemmer, Reza Gheitasi, Raul N. Jamin, Niklas Baerlecken, Maren Winkler, Jens Reimann, Cornelia Kornblum, Claus-Jürgen Bauer, Markus Essler, Valentin S. Schäfer

**Affiliations:** 1 Department of Rheumatology and Clinical Immunology, University Hospital of Bonn, Bonn, Germany; 2 Department of Nuclear Medicine, University Hospital of Bonn, Bonn, Germany; 3 Department of Neuromuscular Diseases, Center for Neurology, University Hospital of Bonn, Bonn, Germany; 4 Department of Cardiology, University Hospital of Bonn, Bonn, Germany

## Abstract

Dermatomyositis (DM) is an autoimmune disease characterised by muscle weakness, myalgia, and extramuscular manifestations and is classified as an idiopathic inflammatory myopathy (IIM). Imaging modalities are important for diagnosis and disease monitoring. Vascular adhesion protein-1 (VAP-1), an inflammation-specific adhesion molecule, may represent a promising target for disease assessment. Recently, the radiotracer [^68^Ga]Ga-DOTA-Siglec-9, which binds to VAP-1, was introduced for visualising vascular inflammation in giant cell arteritis. This case report aimed to generate preliminary insights into the pathophysiological involvement of VAP-1 and the possible diagnostic utility of [^68^Ga]Ga-DOTA-Siglec-9 positron emission tomography/computed tomography (PET/CT) in a patient with DM. A 59-year-old female patient with newly diagnosed, untreated DM was recruited. Demographic data, disease history, and laboratory parameters, including soluble VAP-1 (sVAP-1) levels, were collected. The patient underwent a [^68^Ga]Ga-DOTA-Siglec-9 PET/CT scan, followed by muscle biopsy of the vastus lateralis muscle. The patient’s sVAP-1 level was 416.1 pg/mL, compared to 726.1 ± 126.7 pg/mL in controls (n = 8). The [^68^Ga]Ga-DOTA-Siglec-9 PET/CT scan demonstrated increased tracer uptake in the muscles of the shoulder girdle, back, forearm, and pharynx, consistent with clinical symptoms. Immunohistochemistry demonstrated endothelial VAP-1 expression in perimysial vessels along with DM-specific muscle alterations. In conclusion, we report the first application of [^68^Ga]Ga-DOTA-Siglec-9 PET/CT in a patient with IIM. While the findings support the feasibility of this imaging modality and provide preliminary evidence for the involvement of both soluble and endothelial-bound VAP-1 in immune cell trafficking and inflammation, any conclusions regarding its diagnostic or monitoring utility must remain cautious. Further studies with larger cohorts and longitudinal imaging are needed to evaluate [^68^Ga]Ga-DOTA-Siglec-9 PET/CT as a potential molecular biomarker for inflammation in IIM.

## INTRODUCTION

Idiopathic inflammatory myopathies (IIM) are a heterogeneous group of chronic inflammatory skeletal muscle disorders characterised by progressive proximal muscle weakness, myalgia, and extramuscular manifestations such as pulmonary involvement [1]. These disorders impair quality of life and are associated with increased morbidity and mortality [1,2]. Myositis-specific antibodies aid to stratify patients into subtypes, including dermatomyositis (DM), inclusion body myositis, immune-mediated necrotising myopathy, antisynthetase syndrome, and overlap myositis [3]. The prevalence of IIM ranges from 2 to 25 per 100,000 annually, peaking between 70 to 80 years of age [4].

Imaging technologies are currently not incorporated into the 2017 European League Against Rheumatism (EULAR)/American College of Rheumatology (ACR) classification criteria for myositis, although their utility in diagnosing and monitoring IIM is increasingly recognised [5]. Various modalities, such as ultrasound, magnetic resonance imaging (MRI), and positron emission tomography-computed tomography (PET/CT), are being employed. Imaging with MRI can distinguish active inflammation from chronic damage, identify subtypes, and guide muscle biopsy [6]. Quantitative muscle ultrasound demonstrates good sensitivity and a strong positive predictive value for diagnosing IIM and effectively guides muscle biopsy procedures [6,7]. Whole-body PET/CT with [^18^F]fluorodeoxyglucose ([^18^F]FDG) can detect inflammatory lesions and malignancies, facilitating the exclusion of paraneoplastic processes, particularly in DM [6,8].

Beyond [^18^F]FDG, novel inflammation-specific radiotracers offer potential for improved evaluation of inflammatory, autoimmune, and malignant diseases. Recently, [^68^Ga]Ga-DOTA-Siglec-9 has emerged as a tool for assessing disease activity in giant cell arteritis [9,10]. Sialic acid-binding immunoglobulin-like lectin-9 (Siglec-9), a leukocyte ligand of vascular adhesion protein-1 (VAP-1), is a type II transmembrane sialoglycoprotein [11]. It exists in both the membrane-bound and the soluble forms (sVAP-1), with the latter generated through matrix metalloproteinase (MMP)-mediated cleavage of the membrane-bound counterpart [12]. VAP-1 is expressed highly in vascularised tissues, localising to vascular smooth muscle caveolae and endothelial vesicles [13]. Under inflammatory conditions, driven by tumour necrosis factor (TNF)α, IFN-γ, and interleukin (IL)-1β, VAP-1 translocates to the cell surface, binding Siglec-9 on granulocytes and monocytes [14]. This interaction triggers oxidative deamination, leading to endothelial damage and amplifying inflammation [15]. Additionally, VAP-1 upregulates endothelial adhesion molecules (intercellular adhesion molecule-1, mucosal addressin cell adhesion molecule 1, E-/P-selectin), induces CXCL8 secretion, activates NF-κB, and enhances MMP expression, promoting leukocyte recruitment and transmigration [10,12].

Given the pivotal role of immune cell infiltration and tissue inflammation in IIM, along with the selective upregulation of VAP-1 during inflammation, the involvement of VAP-1 in the pathophysiology of IIM is highly compelling. This case study aimed to explore the potential of [^68^Ga]Ga-DOTA-Siglec-9 PET/CT as a diagnostic tool and to generate hypotheses regarding VAP-1-mediated mechanisms in the pathogenesis of IIM in a newly diagnosed, untreated DM patient.

## CASE REPORT

### Clinical, laboratory, and imaging assessment

A 59-year-old female patient with newly diagnosed, treatment-naïve DM was recruited from the Department of Rheumatology at the University Hospital Bonn, Germany, in March 2024. The diagnosis was made by a board-certified rheumatologist. The patient further fulfilled the 2017 EULAR/ACR classification criteria for myositis [5]. Demographic data, medical history, and immunoserological parameters were recorded. Furthermore, MRI of the lower extremities and an imaging guided muscle biopsy from the right vastus lateralis were performed. To evaluate sVAP-1 levels, an enzyme-linked immunosorbent assay (ELISA) was conducted according to the manufacturer’s protocol. A Quantikine Immunoassay Control Set 754 (R&D Technologies) was used to validate findings. MMP3 measurement was performed according to the AESKU.GROUP protocol. MMP2 and MMP9 measurements were performed using DUOSET ELISA (Bio-Techne) according to the manufacturer’s protocol. Measurements from our patient were compared to those from 8 age-matched (mean age 57.9 ± 9.8 years; 6 female, 2 male) healthy individuals recruited from the same department.

**Figure 1:**
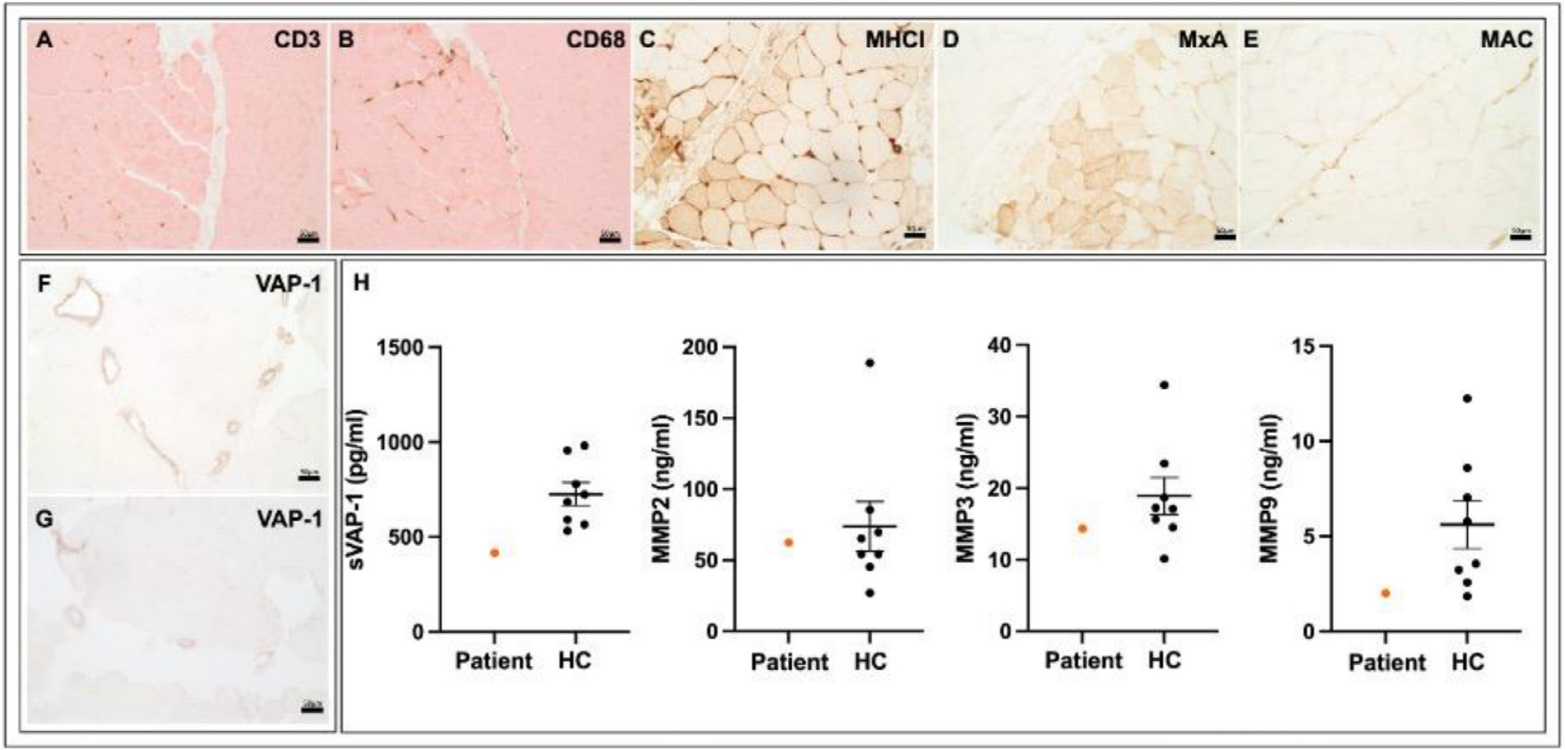
Immunohistochemical staining and enzyme-linked immunosorbent assay results in a patient with dermatomyositis. Immunohistochemical staining of (A) CD3, (B) CD68, (C) MHC-I, (D) MxA, (E) MAC, and (F) VAP-1 in a muscle biopsy of the right vastus lateralis of our patient. Immunohistochemistry revealed prominent endothelial VAP-1 expression in perimysial vessels compared to a noninflammatory control (G). Furthermore, mild perifascicular inflammation, including CD3^+^ and CD68^+^ cells, sarcoplasmic reactivity with anti-MxA, corresponding to anti-MHC-I, and some pathological capillary reactivity with anti-MAC was found. Enzyme-linked immunosorbent assay showed reduced sVAP-1 levels in the dermatomyositis patient compared to a negative control group (n = 8). CD, cluster of differentiation; HC, healthy control; MAC, membrane attack complex; MHC-I, major histocompatibility complex class I; MMP, matrix metalloproteinase; MxA, myxovirus resistance protein A; sVAP-1, soluble vascular adhesion protein-1; VAP-1, vascular adhesion protein-1.

At diagnosis, the patient exhibited progressive, generalised myalgia primarily affecting the shoulder girdle and upper arms, dysphagia, dyspnoea, and typical cutaneous manifestations on the face and hands. Manual muscle testing of 8 muscle groups score was 118/150. C-reactive protein was 1 mg/L, and creatine kinase was 78 U/L. Antinuclear antibody titres were 1:10,240 (nuclear membrane) and 1:5120 (cytoplasmic), with strong positivity for anti-SAE1 and cN1A measured by immunoblotting. The patient’s sVAP-1 level was 416.1 pg/mL, markedly lower than the mean level (726.1 ± 126.7 pg/mL) in our healthy controls . MMP levels were comparable between the patient and controls: MMP-2 (62.5 ng/mL vs 73.7 ng/mL, SD ± 49.6), MMP-3 (14.3 ng/mL vs 18.9 ng/mL, SD ± 7.3), and MMP-9 (2.0 ng/mL vs 5.6 ng/mL, SD ± 3.5). Detailed ELISA results are presented in Figure 1. Muscle MRI revealed oedematous changes consistent with active myositis in the thigh region, such as the right vastus lateralis muscle around the fascia and subfascial areas (Supplementary Fig).

### [^68^Ga]Ga-DOTA-Siglec-9-PET/CT

Radiosynthesis of [^68^Ga]Ga-DOTA-Siglec-9 with 1.32 GBq gallium-68 revealed a decay corrected radiochemical yield of 85.3% (68.8% activity yield). A volume activity of 95.4 MBq/mL and an apparent molar activity of 15.4 MBq/nmol were obtained. Radiochemical purity was >98%. The detailed protocol is available with the full text of this article online (Supplementary Methods).

**Figure 2:**
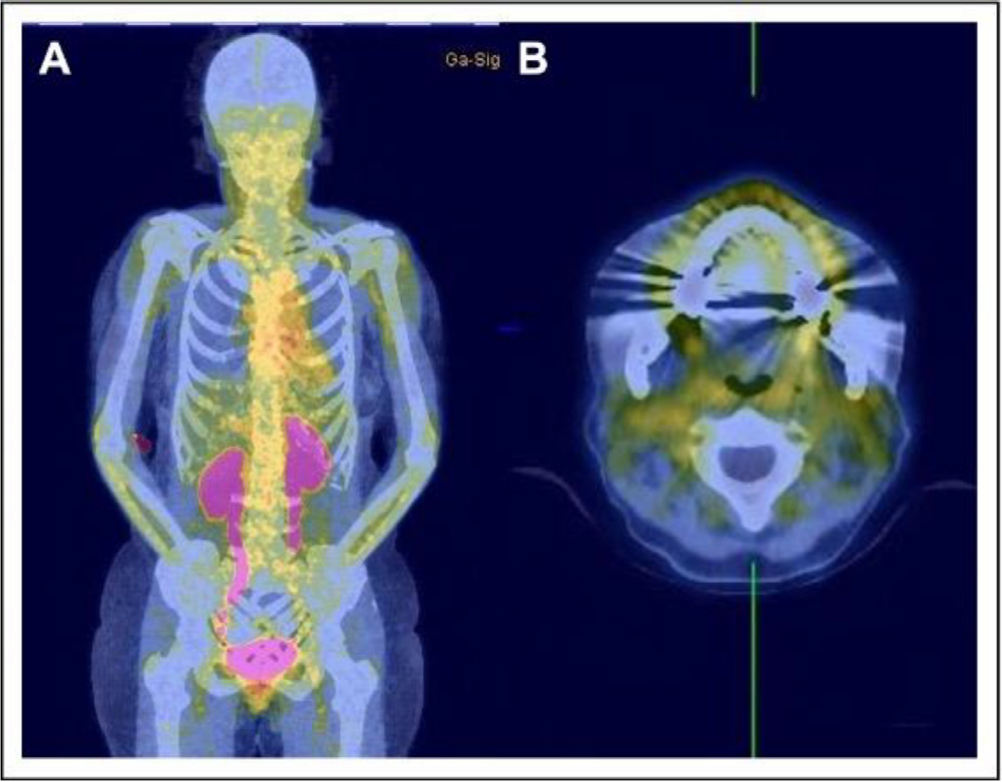
Increased uptake of [^68^Ga]Ga-DOTA-Siglec-9 in various regions in a patient with dermatomyositis on PET imaging. Whole-body PET imaging of a patient with dermatomyositis following intravenous injection of 123 MBq [^68^Ga]Ga-DOTA-Siglec-9. Radiotracer distribution, assessed 50 minutes postinjection, revealed increased uptake in the muscles of the shoulder girdle, back, forearms, as well as the tongue and pharynx. PET, positron emission tomography.

The patient underwent [^68^Ga]Ga-DOTA-Siglec-9 PET/CT, receiving an injection of 123 MBq of the radiotracer. The injection of [^68^Ga]Ga-DOTA-Siglec-9 injection was well tolerated. Fifty minutes postinjection, a low-dose CT was performed for attenuation correction, followed by a whole-body PET scan (1 min/bed). Imaging with [^68^Ga]Ga-DOTA-Siglec-9 PET/CT revealed increased tracer uptake in the musculature of the shoulder girdle, back, forearm, tongue, and pharynx (Fig 2). No signs of malignancy were observed. The radiotracer injection was well tolerated. The protocol is available with the full text of this article online (Supplementary Methods).

### Immunohistochemical staining

Immunohistochemical analysis of the vastus lateralis muscle biopsy revealed perifascicular upregulation of major histocompatibility complex (MHC) class I. Myxovirus resistance protein A (MxA) reactivity was observed in some perifascicular fibres, colocalising with MHC-I-positive areas. Mild endomysial inflammatory infiltrates were present, characterised by increased cluster of differentiation (CD)3-positive T cells and CD68-positive macrophages, predominantly in perifascicular regions. Anti-membrane attack complex (MAC) staining showed slight capillary reactivity, while MHC-II expression appeared almost normal. Although perimysial involvement was prominent, the presence of MHC-II and MxA argued against immune myopathy with perimysial pathology. Additionally, immunohistochemistry demonstrated increased endothelial VAP-1 expression in perimysial vessels compared to a noninflammatory control (Fig 1).

## DISCUSSION

This exploratory case report is the first to assess [^68^Ga]Ga-DOTA-Siglec-9 PET/CT as a biomarker for noninvasive molecular imaging in IIM and to investigate the pathophysiological role of VAP-1 in DM pathogenesis.

Muscle biopsy revealed perifascicular damage with MHC-I upregulation and MxA positivity, hallmark features of interferon-driven DM pathology. Mild, predominantly perifascicular endomysial inflammation was characterised by CD3^+^ T cells and CD68-positive macrophages, indicative of immune-mediated damage via cellular and humoral mechanisms. Anti-MAC reactivity in perifascicular capillaries suggested a link between DM pathogenesis, microangiopathy, and vascular inflammation. Furthermore, immunohistochemistry revealed endothelial VAP-1 expression in perimysial vessels corresponding to [^68^Ga]Ga-DOTA-Siglec-9 PET/CT tracer uptake in clinically affected regions, reinforcing evidence of vascular inflammation.

However, the association between inflammation in IIM, particularly DM, and VAP-1 expression warrants further exploration. In IIM, inflammatory sites are characterised by infiltration of inflammatory immune cells including macrophages and T cells, in part driven by elevated levels of IFN-γ, IL-1β, and TNFα [1,16]. While traditional models of DM pathogenesis emphasised complement activation and CD4^+^ T cell pathways, latest evidence indicates a more complex interplay involving CD8^+^ cells and MHC-I upregulation [17,18]. This proinflammatory cytokine milieu aligns with the selective role of VAP-1 in mediating CD8^+^ T cell adhesion to high endothelial venules and endothelial cells [19].

The reduced sVAP-1 levels raise critical questions. The discrepancy between increased endothelial VAP-1 alongside lower sVAP-1 levels suggests diminished cleavage of endothelial-bound VAP-1 by MMPs. Although the pathophysiological role of MMPs, such as MMP-2 and MMP-9, has primarily been linked to overexpression in muscle fibres and autoinvasive CD8^+^ cells during acute inflammation, facilitating lymphocyte migration [20], this finding points to additional complexity. One possible explanation involves upregulation of endogenous MMP inhibitors, impairing VAP-1 cleavage despite elevated MMP levels. Alternatively, reduced enzymatic activity of MMPs may limit sVAP-1 cleavage, or MMP activity may shift towards extracellular matrix remodelling rather than VAP-1 cleavage, reflecting altered enzyme localisation and function. Chronic inflammatory signalling could further downregulate sVAP-1 cleavage. Further investigation with larger cohorts in different disease states is required to determine whether reduced sVAP-1 cleavage mitigates disease progression through the loss of monoamine oxidase activity in human blood or exacerbates it by prolonging endothelial VAP-1 expression, thereby sustaining inflammation and vascular injury.

**Figure 3:**
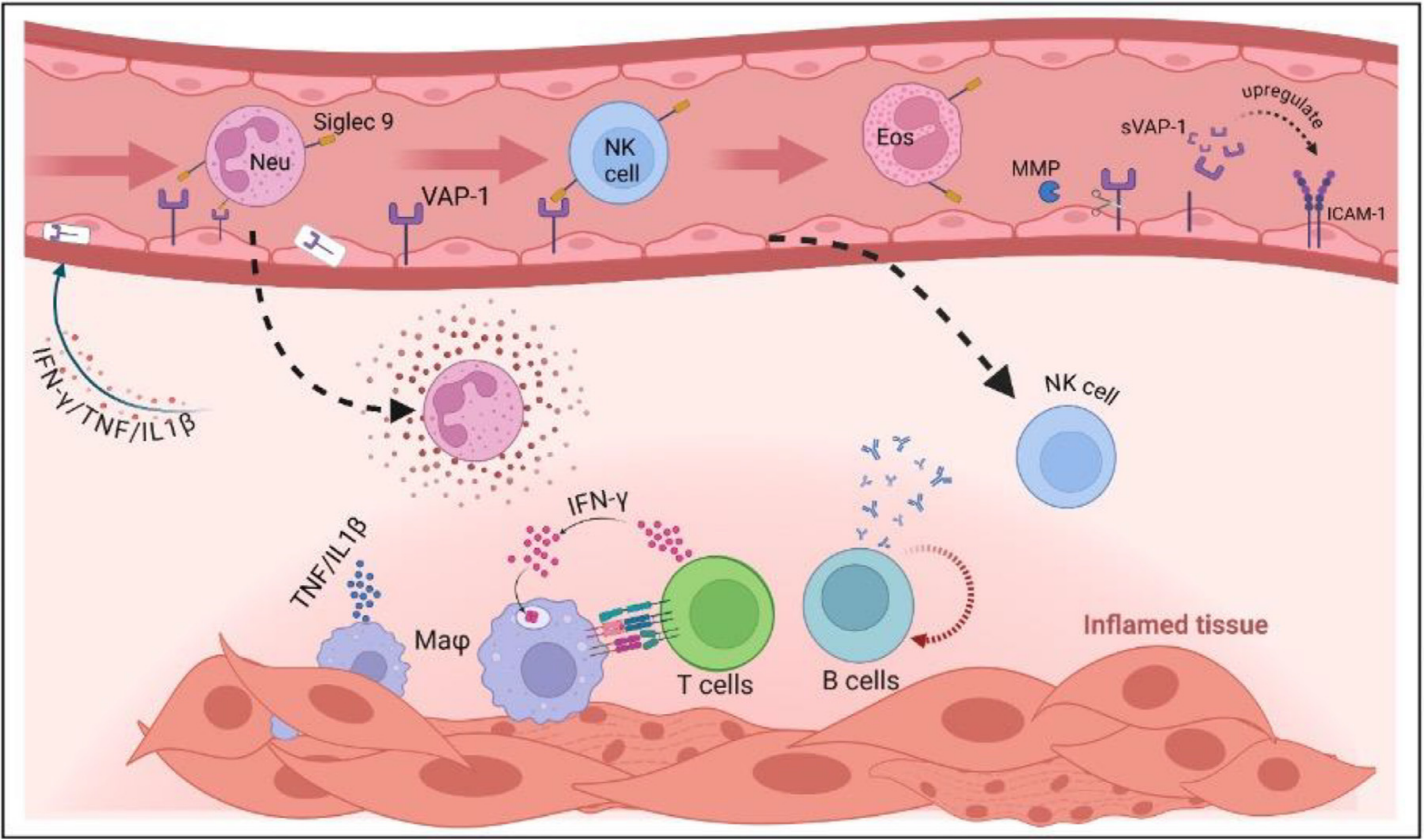
Pathophysiological role of VAP-1 in immune cell migration and inflammation in idiopathic inflammatory myopathies. Schematic illustration of the role of VAP-1 in immune cell migration and inflammation in idiopathic inflammatory myopathies. Inflammatory stimuli (eg, IFN-γ, TNF-α, IL-1β) induce VAP-1 translocation to the cell surface, where it binds Siglec-9 (eg, on neutrophils), facilitating leukocyte recruitment and transmigration. VAP-1 further upregulates endothelial adhesion molecules (ICAM-1, MAdCAM-1, E-/P-selectin) and enhances MMP expression, which cleaves sVAP-1. Created with BioRender.com. Eos, eosinophil; ICAM-1, intercellular adhesion molecule 1; IFN, interferon; IL, interleukin; MAdCAM-1, mucosal addressin cell adhesion molecule 1; Maφ, macrophage; MMP, matrix metalloproteinase; Neu, neutrophil; NK, natural killer; Siglec-9, sialic acid-binding immunoglobulin-like lectin-9; sVAP-1, soluble vascular adhesion protein-1; TNF, tumour necrosis factor; VAP-1, vascular adhesion protein-1.

To summarise, this case report is the first to introduce [^68^Ga]Ga-DOTA-Siglec-9 PET/CT and VAP-1 as a novel molecular imaging approach in IIM. The inflammation-specificity of [^68^Ga]Ga-DOTA-Siglec-9 PET/CT may provide complementary information in the diagnostic evaluation of IIM. Our findings suggest a potential pathophysiological involvement of both soluble and endothelial-bound VAP-1 in immune cell trafficking and inflammatory processes in IIM (Fig 3). Future studies involving larger patient cohorts and a variety of IIM subtypes are needed to further investigate the diagnostic and prognostic relevance of VAP-1 and to elucidate its underlying biological role. Longitudinal imaging will be essential to assess whether [^68^Ga]Ga-DOTA-Siglec-9 uptake and sVAP-1 levels correlate with clinical disease activity, laboratory markers, and treatment response over time and to validate [⁶⁸Ga]Ga-DOTA-Siglec-9 PET/CT as a molecular biomarker for inflammation in IIM.

## Competing interests

All authors declare they have no competing interests.

## Appendix Supplementary materials

Image, application 1

Image, application 2

## Footnotes

Supplementary material associated with this article can be found in the online version at doi:10.1016/j.ero.2025.04.005.

## References

[1] Dalakas MC. (2015). Inflammatory muscle diseases. N Engl J Med.

[2] Sun K.Y., Fan Y., Wang Y.X., Zhong Y.J., Wang GF. (2021). Prevalence of interstitial lung disease in polymyositis and dermatomyositis: a meta-analysis from 2000 to 2020. Semin Arthritis Rheum.

[3] Basuita M., Fidler LM. (2022). Myositis antibodies and interstitial lung disease. J Appl Lab Med.

[4] Khoo T., Lilleker J.B., Thong B.Y.H., Leclair V., Lamb J.A., Chinoy H. (2023). Epidemiology of the idiopathic inflammatory myopathies. Nat Rev Rheumatol.

[5] Lundberg I.E., Tjärnlund A., Bottai M., Werth V.P., Pilkington C., Visser M. (2017). 2017 European League Against Rheumatism/American College of Rheumatology classification criteria for adult and juvenile idiopathic inflammatory myopathies and their major subgroups. Ann Rheum Dis.

[6] Zubair A.S., Salam S., Dimachkie M.M., Machado P.M., Roy B. (2023). Imaging biomarkers in the idiopathic inflammatory myopathies. Front Neurol.

[7] Gazeley D.J., Cronin M.E. (2011). Diagnosis and treatment of the idiopathic inflammatory myopathies. Ther Adv Musculoskelet Dis.

[8] Parida G.K., Roy S.G., Kumar R. (2017). FDG-PET/CT in skeletal muscle: pitfalls and pathologies. Semin Nucl Med.

[9] Petzinna S.M., Küppers J., Schemmer B., Kernder A.L., Bauer C.J., von der Emde L. (2024). Case report: detecting giant cell arteritis in [^68^Ga]Ga-DOTA-Siglec-9-PET/CT. Front Immunol.

[10] Petzinna S.M., Bauer C.J., Schäfer VS. (2024). Vascular-adhesion protein 1 in giant cell arteritis and polymyalgia rheumatica. Front Med (Lausanne).

[11] Aalto K., Autio A., Kiss E.A., Elima K., Nymalm Y., Veres T.Z. (2011). Siglec-9 is a novel leukocyte ligand for vascular adhesion protein-1 and can be used in PET imaging of inflammation and cancer. Blood.

[12] Salmi M., Jalkanen S. (2019). Vascular adhesion protein-1: a cell surface amine oxidase in translation. Antioxid Redox Signal.

[13] Solé M., Unzeta M. (2011). Vascular cell lines expressing SSAO/VAP-1: a new experimental tool to study its involvement in vascular diseases. Biol Cell.

[14] Virtanen H., Autio A., Siitonen R., Liljenbäck H., Saanijoki T., Lankinen P. (2015). ^68^Ga-DOTA-Siglec-9–a new imaging tool to detect synovitis. Arthritis Res Ther.

[15] Heuts D.P.H.M., Gummadova J.O., Pang J., Rigby S.E.J., Scrutton N.S. (2011). Reaction of vascular adhesion protein-1 (VAP-1) with primary amines: mechanistic insights from isotope effects and quantitative structure-activity relationships. J Biol Chem.

[16] Tabata M.M., Hodgkinson L.M., Wu T.T., Li S., Huard C., Zhao S. (2023). The type I interferon signature reflects multiple phenotypic and activity measures in dermatomyositis. Arthritis Rheumatol.

[17] Bender A., Ernst N., Iglesias A., Dornmair K., Wekerle H., Hohlfeld R. (1995). T cell receptor repertoire in polymyositis: clonal expansion of autoaggressive CD8+ T cells. J Exp Med.

[18] Dalakas M.C., Hohlfeld R. (2003). Polymyositis and dermatomyositis. Lancet.

[19] Salmi M., Hellman J., Jalkanen S. (1998). The role of two distinct endothelial molecules, vascular adhesion protein-1 and peripheral lymph node addressin, in the binding of lymphocyte subsets to human lymph nodes. J Immunol.

[20] Kieseier B.C., Schneider C., Clements J.M., Gearing A.J., Gold R., Toyka K.V. (2001). Expression of specific matrix metalloproteinases in inflammatory myopathies. Brain.

