# Supplementary material for: Molecular imaging and immune cell adhesion via vascular adhesion protein-1 in idiopathic inflammatory myopathy: a case report
Source: EULAR Rheumatol Open. 2025 May 10;1(2):34–8. doi: 10.1016/j.ero.2025.04.005 (PMC13292238; doi:10.1016/j.ero.2025.04.005)
Supplement: Supplementary file 2 [file mmc2.docx]

*
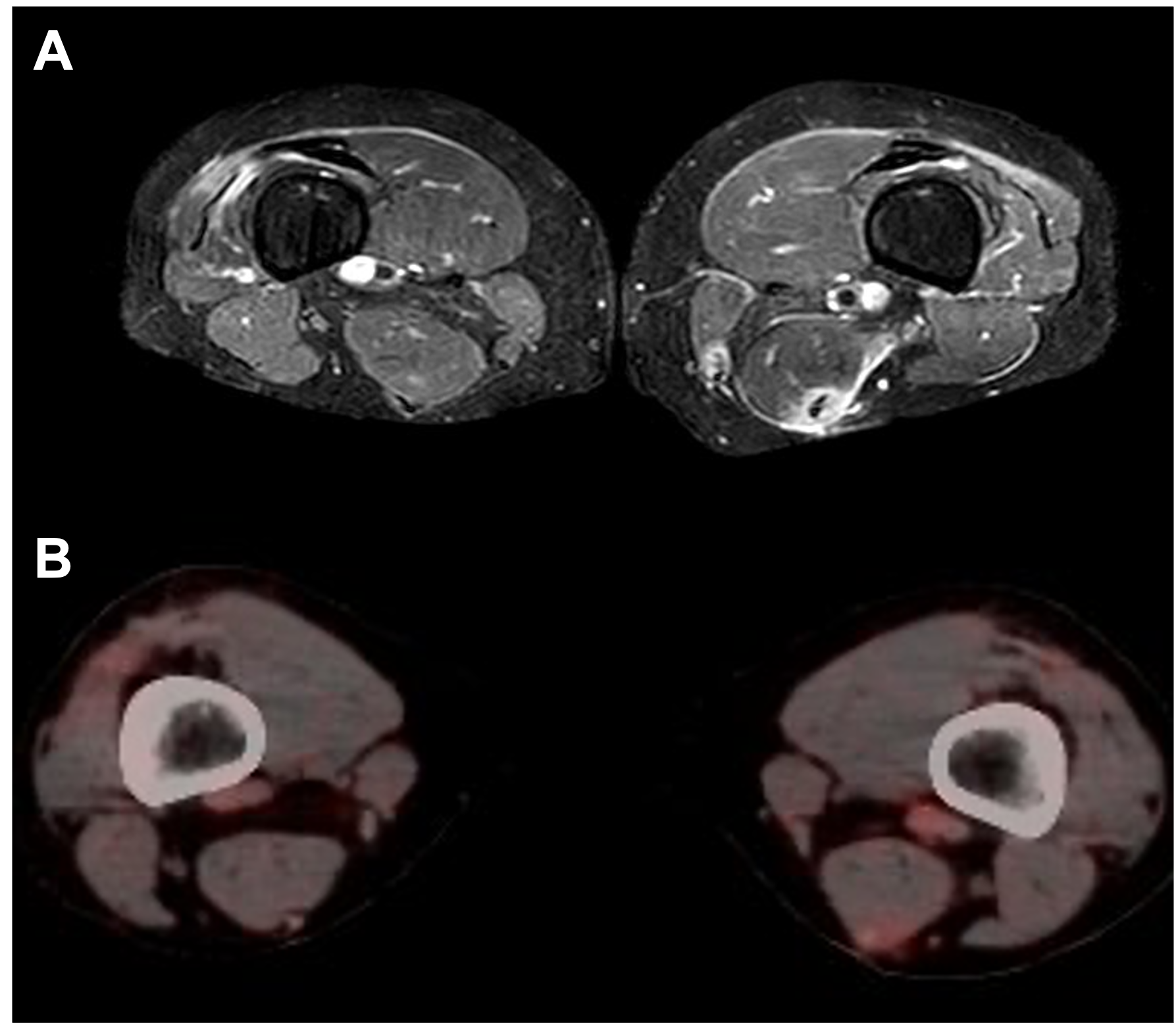
*

*sFigure 1: Cross-sectional MRI and corresponding [⁶⁸Ga]Ga-DOTA-Siglec-9 PET/CT of Thighs in a Patient with Dermatomyositis*

*T2-weighted MRI of the thighs (A) shows perifascial and subfascial edema in the vastus lateralis muscle, consistent with active inflammation. Based on these findings, the site for subsequent muscle biopsy was selected in accordance with established MRI-guided standard operating procedures to ensure optimal diagnostic yield. The corresponding [⁶⁸Ga]Ga-DOTA-Siglec-9 PET/CT image (B) reveals mild but discernible tracer uptake in the same region. Abbreviations****:*** *CT = Computed tomography, MRI = Magnetic resonance imaging, PET = Positron Emission Tomography.*
